# Cerebral mechanism of celecoxib for treating knee pain: study protocol for a randomized controlled parallel trial

**DOI:** 10.1186/s13063-018-3111-8

**Published:** 2019-01-16

**Authors:** Chenjian Tang, Xiaohui Dong, Wenhua He, Shirui Cheng, Yang Chen, Yong Huang, Bao Yin, Yu Sheng, Jun Zhou, Xiaoli Wu, Fang Zeng, Zhengjie Li, Fanrong Liang

**Affiliations:** Acupuncture and Tuina School, The 3rd Teaching Hospital, Chengdu University of Traditional Chinese Medicine, No. 37, Twelve Bridges Road, Jinniu District, Chengdu, 610075 China

**Keywords:** Celecoxib, fMRI, Chronic pain, Knee osteoarthritis, Central mechanism

## Abstract

**Background:**

Celecoxib is frequently prescribed to treat knee osteoarthritis (KOA), but how celecoxib influences the activity of the central nervous system to alleviate chronic pain remains unclear.

**Methods:**

One hundred eight patients with KOA will be enrolled in this study. Patients will be allocated randomly to three groups: the celecoxib group, the placebo group, and the waiting list group. The patients in the celecoxib group will orally take celecoxib 200 mg once daily and the patients in placebo group with placebo 200 mg every day for 2 weeks. Functional magnetic resonance imaging scan will be performed on all patients at baseline and the end of interventions to detect the cerebral activity changes. The short form of McGill pain questionnaire and the Visual Analog Scale will be used as the primary endpoints to evaluate the improvement of knee pain. The secondary endpoints include the Western Ontario and McMaster osteoarthritis index (WOMAC), the Attention Test Scale, the Pain Assessment of Sphygmomanometer, the Self-rating Anxiety Scale, the Self-rating Depression Scale, and 12-Item Short Form Health Survey (SF-12).

**Discussion:**

The results will investigate the influence of celecoxib treatment on cerebral activity of patients with KOA and the possible relationship between the cerebral activity changes and improvement of clinical variables so as to explore the central mechanism of celecoxib in treating knee pain.

**Trial registration:**

ChiCTR-IOR-17012365. Registered on August 14, 2017.

**Electronic supplementary material:**

The online version of this article (10.1186/s13063-018-3111-8) contains supplementary material, which is available to authorized users.

## Background

Non-steroidal anti-inflammatory drugs (NSAIDs), known as cyclooxygenase (COX) inhibitors, are often prescribed for the management of chronic and acute pain worldwide. NSAIDs exert analgesic effects by inhibiting COX activity in the arachidonic acid cascade and thus inhibit prostaglandin (PG) synthesis [1], which leads to a reduction or reversal of peripheral sensitization. Based on the results of animal studies, NSAIDs could also modulate pain intensity by suppressing prostanoid formation in the spinal cord and brain [2, 3], thus affecting central sensitization. On human beings, the peripheral action of NSAIDs for pain management had been well investigated. For instance, Gallelli et al. found that diclofenac, ibuprofen, and celecoxib can decrease tumor necrosis factor-alpha (TNF-α), interleukin-6 (IL-6), and vascular endothelial growth factor in the synovial fluid of the osteoarthritic joint with a parallel improvement in joint pain and function of patients [4]. However, the central action of NSAIDs is less studied, and the majority of these studies performed on healthy subjects or centered on acute pain condition. For example, neuroimaging studies found that instant NSAIDs could attenuate the activity on pain processing cerebral structures in healthy subjects [5] and top-down modulatory circuits in acute pain condition [6]. How the NSAID drugs acting on the central nervous system (CNS) in patients with chronic pain remains unclear and is worthy of further investigation.

Celecoxib, as the first COX-2 selective inhibitor, was introduced into clinical practice nearly 20 years ago [7–9] and widely used for many pain conditions for fewer side effects. It was reported that, compared with other NSAIDs such as ibuprofen and diclofenac at standard dosages, celecoxib was associated with a lower incidence of symptomatic ulcers and ulcer complications and other clinically important toxic effects [10].

As a high prevalence chronic knee pain [11], the prevalence of radiographic knee osteoarthritis was 19% and 28% among adults over 45 years old in Framingham study and Johnston County Osteoarthritis Project [12]. With the aging of the population, most patients with KOA visit a doctor for their pain treatment [13, 14]. KOA has become the leading cause of disability in the world and brings an increasingly high social and economic burden [15, 16]. Meta-analysis has already indicated that celecoxib treatment (200 mg orally once daily) led to significant improvement in the pain and knee function of patients with osteoarthritis [17]. Although celecoxib treatment is effective for pain management in KOA, little is known about how celecoxib treatment modulates the activity of CNS to alleviate pain in patients with KOA.

With the application of neuroimaging techniques in pain research, investigators not only mapped the brain regions which are involved in the central pathogenesis of chronic pain but also can explore the cerebral responses elicited by analgesics so as to illuminate the central mechanism. With resting-state functional magnetic resonance imaging (rs-fMRI), investigators found that the patients with chronic pain demonstrated the alternations in functional connectivity (FC) of medial prefrontal-default mode network and the regional homogeneity (ReHo) value changes of anterior cingulate cortex [18–21].

These results greatly enhance the understanding of the central mechanism of chronic pain and provide a new approach to the research and development of drugs. For instance, Xiaoyan Chen et al. had shown that acupuncture can regulate the descending pain modulatory system of patients with KOA by enhancing the FC between the right frontoparietal network and medial prefrontal cortex [22].

So this trial aims to (1) investigate the influence of celecoxib treatment on the brain activities of patients with KOA compared with that of placebo treatment and waiting list by fMRI and (2) analyze the possible correlations between the changes of cerebral activity and the improvement of clinical variables in each group so as to explore how the celecoxib manages pain by modulating brain function to treat KOA.

## Design and methods

### Study design

**Fig. 1:**
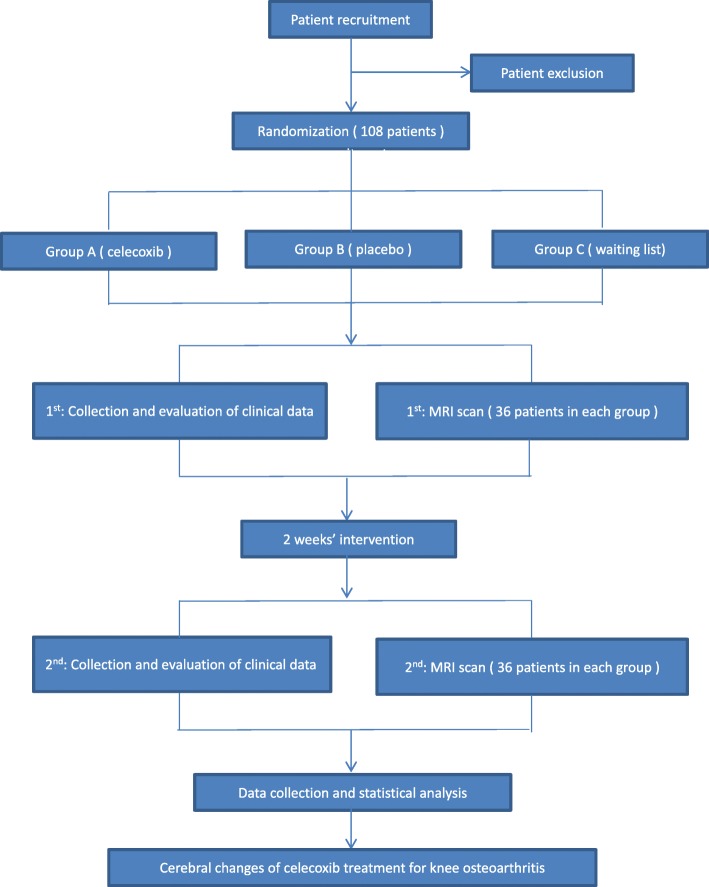
Flowchart of the trial. Abbreviation: *MRI* magnetic resonance imaging

This is a clinical neuroimaging study aiming to investigate the central mechanism of how celecoxib alleviates knee pain for treating KOA. A total of 108 patients with KOA will be randomly allocated to three different groups. Each group includes 36 patients with KOA. The three groups are group A (celecoxib administrated orally once a day), group B (placebo administrated orally once a day), and group C (waiting list). The treatment period will last for 2 weeks. Outcome measurements and MRI scan will be assessed at baseline and at the end of treatment. The changes of clinical variables and cerebral activity of each group will be analyzed after data collection (Fig. 1).

### Participants

Knee pain patients whose KOA is diagnosed according to American College of Rheumatology (ACR) criteria (1991 revised version) [23] will be recruited from outpatient or inpatient departments of the First Affiliated Hospital of Chengdu University of Traditional Chinese Medicine (TCM) and the Third Affiliated Hospital of Chengdu University of TCM. Potential patients will also be recruited through posters, internet, and leaflets.

### Inclusion criteria

Inclusion criteria require that the patients (1) match the diagnosis criteria for KOA set by ACR in 1991 [23], (2) are between 40 and 60 years of age and are right-handed, (3) have not taken any pain killer medicine within 1 month, (4) have at least 3 months of knee pain duration, (5) have an average score on the knee pain Visual Analog Scale (VAS) of at least 3 cm (range of 0 to 10 cm) in the past 2 weeks, (6) have knee joint radiological degree of 0–II in accordance with the Kellgren–Lawrence scale [24], and (7) have signed a written informed consent form.

### Exclusion criteria

Patients will be excluded if they (1) are alcohol or drug abusers or abuse other medications that may influence brain imaging outcomes; (2) are pregnant or lactating; (3) have psychiatric, neurologic, gastrointestinal, cardiovascular, infectious, immunologic, respiratory, or renal illnesses; (4) have any other chronic pain conditions or a history of head trauma with loss of consciousness; (5) had diagnosed rheumatoid arthritis or other leg-related pain disorders; (6) have MRI contraindications such as claustrophobia, cardiac pacemaker, defibrillator, heart stenting, or intrauterine device; (7) have active peptic ulcer or a history of peptic ulcer; or (8) are allergic to celecoxib.

### Sample size

The sample size calculation of neuroimaging study is different from that of classic randomized controlled trials. The neuroimaging study focuses on investigating mechanism but not evaluating efficacy. General speaking, in MRI studies, 12 to 15 patients per group provide a statistical result [24, 25]. Our previous literature study shows that at least 20 patients per group can achieve stable results for brain functional network analysis [26]. In this study, we require 30 patients per group in this trial. However, considering a 20% dropout rate and possible excessive head motion during scanning, we will include 36 participants with KOA in each group. Finally, we plan to enroll 108 participants and each group will undergo MRI scans twice to investigate the different central responses among celecoxib, placebo, and waiting list treatment in knee pain KOA patients.

### Informed consent

This study protocol has been approved by the supervision of the Sichuan Regional Ethics Review Committee on TCM (ethical approval number 2016KL-017) and been registered at Chinese Clinical Trial Registry (registration number ChiCTR-IOR-17012365). The authors retain full control of the article’s content. All patients will be informed of the random allocation of celecoxib, placebo, or waiting list treatment and the possible risks. They will voluntarily sign an informed consent form before enrollment. The patients will be free to withdraw from the study at any time without a specific reason and without any penalty or loss of benefits. However, we will attempt to understand the reason for withdrawal and encourage the participant to remain in the study if possible.

### Patient safety

**Fig. 2:**
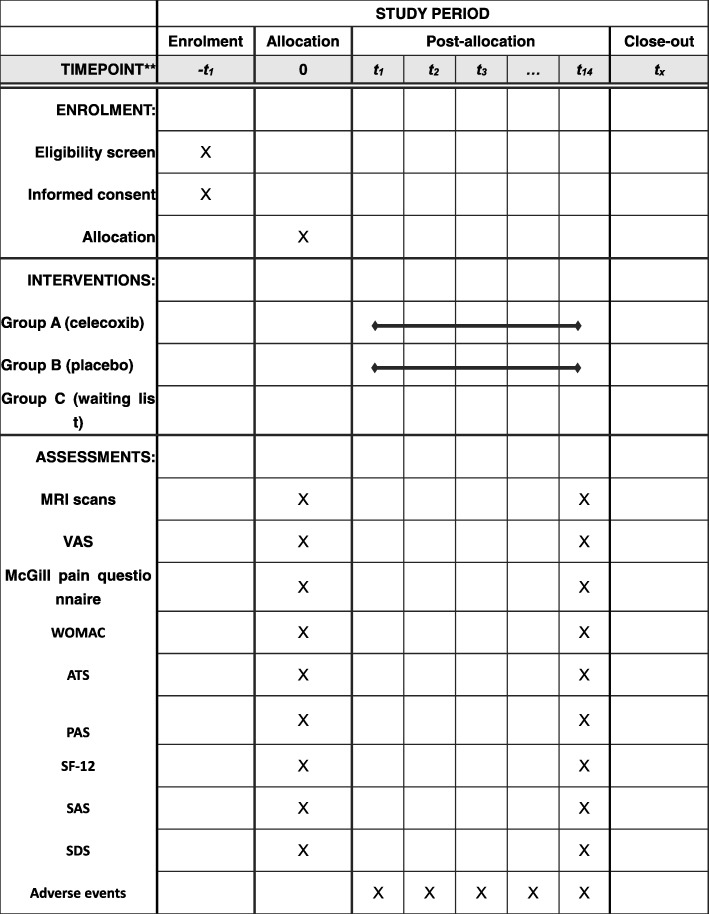
Schedule of enrollment, interventions, and assessments. Abbreviations: *ATS* Attention Test Scale, *MRI* magnetic resonance imaging, *PAS* Pain Assessment of Sphygmomanometer, *SAS* Self-rating Anxiety Scale, *SDS* Self-rating Depression Scale, *SF-12* 12-Item Short Form Health Survey, *WOMAC* Western Ontario and McMaster osteoarthritis index

This study will be performed in accordance with the guidance and principles of the Declaration of Helsinki. Adverse events caused by celecoxib, such as nausea, vomiting, or other severe events, will be processed immediately and recorded in detail in the case report forms. Each patient will get paid for study participation. Figure 2 provides a complete overview of the time schedule of the enrollment, interventions, and assessments.

### Randomization

Eligible patients will be randomly assigned in a ratio of 1:1:1 to the celecoxib group, placebo group, and waiting list group with 36 patients in each group. Random number lists will be created in accordance with PROC PLAN of SAS 9.2 (SAS Institute Inc., Cary, NC, USA). The randomization allocation will use sequentially numbered, opaque, and sealed envelopes by an independent assistant. The celecoxib group and the placebo group will be concealed from the researchers until completion of the statistical analysis.

### Blinding

As waiting list group without any medical intervention, while the other two groups will be given celecoxib or placebo 200mg daily, it is impossible to blind the patients, clinicians and investigators totally. Patients and researchers of the celecoxib group and the placebo group will be unaware of the assignments until completion of the statistical analysis. Only an independent researcher in the pharmacy department knows the allocation of the celecoxib and placebo groups. The evaluators and statisticians will be blinded to the group allocation during outcome evaluation and data analysis to reduce the risk of bias.

### Interventions

Patients in the celecoxib group will orally take a celecoxib capsule (approval number J20030098, manufactured by Pfizer Pharmaceutical Co. Ltd., Beijing, China) at a daily dosage of 200 mg for 2 weeks. Patients in the placebo group will receive a placebo capsule (manufactured by pharmacy department of Affiliated Hospital of Chengdu University of TCM) with oral administration at the same dosage as celecoxib capsule once a day for 2 weeks. The placebo capsule is identical in appearance, smell, and texture to the celecoxib capsule. Patients in the waiting list group will be observed for 2 weeks without any medical or other interventions. After 2 weeks’ observation, patients in the waiting list group and placebo group could choose either 2 weeks’ celecoxib treatment or acupuncture treatments free of charge.

### Concomitant medications

Patients will be instructed not to take any caffeine (such as tea and coffee) or have any other treatments for KOA during the study. In case of severe knee pain, ibuprofen (300 mg each capsule with sustained release) will be allowed as rescue medication and should be recorded in the knee pain diary. Patients are also asked to record the name, dose, date, and the exact time of the medications used and reported to the researcher if they take any regular medications during the trial.

### MRI data acquisition

MRI data will be acquired with a 3.0 T magnetic resonance scanner (GE 3.0 T MR750; GE Healthcare, Chicago, IL, USA) with an eight-channel phase-array head coil at the MRI Center in University of Electronic Science and Technology of China. Subjects will be asked to stay awake and to keep their heads still during the scan, with their eyes closed and ears plugged. Subjects will be asked to keep relaxed and not think anything particular during the whole scan. MRI scans will be assessed twice at baseline and after 2 weeks’ intervention or observation period for each patient.

Prior to the rs-fMRI scan, a high-resolution structural image for each subject will be acquired by using a three-dimensional MRI sequence (3DT1) with a voxel size of 1 mm^3^ employing an axial fast spoiled gradient recalled sequence (repetition time 6.008 ms, echo time 1.7 ms, data matrix 256 × 256, field of view 256 × 256 mm^2^). The rs-fMRI images will be obtained with echo-planar imaging (31 contiguous slices with a slice thickness of 5 mm, repetition time 2000 ms, echo time 30 ms, flip angle 90°, field of view 240 × 240 mm^2^, data matrix 64 × 64, total volumes 205). After the rs-fMRI scan, diffusion tensor imaging sequences with single-shot echo planar imaging will be acquired to detect the possible abnormality of the white matter with the following parameters: field of view 256 × 256 mm^2^, repetition time 8500 ms, echo time minimum, matrix = 128 × 128, slice thickness 2 mm, and 78 continuous axial slices with no gap. Two diffusion-weighted sequences will be acquired by using gradient values b = 0 s/mm^2^ and b = 1000 s/mm^2^ with the diffusion-sensitizing gradients applied along 64 non-linear directions.

### Outcome measurement

The clinical evaluations will comprise four components: knee pain, knee function, cognition, and emotion. All evaluations will be performed twice: at baseline and after 2 weeks’ intervention or observation. All evaluations will be evaluated by two trained licensed physicians.

The primary outcome measurements are VAS and the short form of the McGill pain questionnaire of average knee pain in the last 2 weeks and the VAS of knee pain during the MRI scan. The short form of the McGill pain questionnaire could measure the sensory and affective component of pain.

The secondary outcome measurements are the Western Ontario and McMaster Universities Osteoarthritis Index (WOMAC) [27], Attention Test Scale (ATS), Pain Assessment of Sphygmomanometer, Self-rating Anxiety Scale (SAS), Self-rating Depression Scale (SDS), and 12-Item Short Form Health Survey (SF-12). WOMAC could help measure the pain, knee joint function, and quality of life of patients with KOA.

### Data management

The case report form includes observation time points, scanning time points, outcome measures, adverse events, and safety evaluations. The researchers will be required to follow the requirements of the case report form and fill in the relevant information in a timely and accurate manner. Data collection will be performed by study physician, and the quality of the data will be supervised by two independent experienced clinical researchers.

### Data analysis

Before the data analyses, the research group will provide a statistical scheme to the statisticians. The scheme will include the required data and processing method. The data will be processed and analyzed by the statisticians in accordance with the scheme.

For the clinical data, statistical analyses will be performed with SPSS 21.0 statistics software (IBM Corporation, Armonk, NY, USA). The data analysis process will be completed by statisticians who are independent from the research team and blinded to the test settings. A *t* test will be used to compare the numerical variables in the within-group analyses, including the VAS, short form of the McGill pain questionnaire, WOMAC, ATS, SAS, SDS, Pain Assessment of Sphygmomanometer (PAS), and SF-12. Analysis of variance and the Kruskal–Wallis test will be used for numerical variables in the between-group analyses, including age, height, weight, scores of VAS, short form of the McGill pain questionnaire, WOMAC, ATS, SAS, SDS, PAS, and SF-12. The chi-squared test will be used for categorical variables. A two-sided test is applied for available data, and a *P* value of less than 0.05 is considered statistically significant.

The fMRI scan data will be preprocessed and analyzed by SPM12 software (http://www.fil.ion.ucl.ac.uk/spm/) performed on MATLAB 2015b (MathWorks, Inc., Natick, MA, USA). The preprocessing steps will include slice timing correction, head motion correction, spatial normalization, spatial smoothing, and detrending. After data preprocessing, amplitude of low-frequency fluctuation, ReHo, and FC will be used to investigate the cerebral responses of different study groups. Two-sample *t* tests will be used to evaluate possible cerebral responses in each group by within-group analysis (post-treatment minus pre-treatment). We will use between-group analysis to compare the difference in cerebral response changes. Correlation analysis will be conducted to investigate changes between fMRI image data and corresponding clinical data in each group.

## Discussion

This fMRI trial is the first one which focuses on the central mechanism of celecoxib for treating KOA. The results will enhance our understanding of how NSAIDs alleviate pain and the central mechanism of KOA.

Cyclooxygenase can be subdivided into two isoenzymes: cyclooxygenase-1 (COX-1) and cyclooxygenase-2 (COX-2) [7]. Celecoxib, one of the NSAIDs which selectively inhibit COX-2, has been extensively used in the treatment of chronic pain such as osteoarthritis and rheumatoid arthritis [28]. The anti-inflammatory and analgesic effects of celecoxib are known to involve a complex biological process related to multiple pathways, targets, and factors. By restraining COX-2 activity in the arachidonic acid cascade, celecoxib inhibits PG synthesis which can provide anti-inflammatory, antipyretic, and analgesic effects. For example, Funakoshi-Tago et al. have shown that, in a peripheral injury, celecoxib can inhibit the activation of TNF-α–dependent NF-kappa B and produce strong anti-inflammatory activity [29]. In addition, celecoxib can inhibit the neurotoxic activity of macrophages and glial cells in neurons *in vitro* [30]. Demb et al. found that celecoxib had rapid penetration in the CNS in humans and sufficiently concentrated to inhibit COX-2 and prostanoid activity [31]. However, the responses of cerebral activity to celecoxib treatment for chronic pain in humans are less studied and the central mechanism remains uncovered.

With the advantages of high quality of time and spatial resolution, without radiation, quick imaging velocity, and relative lower price [32, 33], fMRI becomes the most commonly used neuroimaging techniques in clinic and research. Compared with task-related fMRI, rs-fMRI has the advantage of providing more comprehensive information on the functional architecture of the brain [34]. In recent years, with rs-fMRI, investigators had investigated the functional alternations in CNS in multiple patients with chronic pain, including migraine and low back pain [32, 35]. Furthermore, using rs-fMRI, investigators explored the cerebral responses to medical interventions in patients with chronic pain. For example, our previous rs-fMRI study on patients with migraine found that acupuncture could influence the ReHo values of multiple regions in the default model network, including posterior cingulate [36]. Jian kong and his collegues using rs-fMRI found that acupuncture treatment could significantly enhance the connectivity of the descending pain modulation pathway major through connection with the posterior medial prefrontal cortex in patients with KOA [37]. These studies show the wide application of rs-fMRI in pain research.

Straight quality control measurements are conducted to avoid bias in this trial to improve the reliability of results. First, at the stage of participant enrollment, we establish rigorous inclusion and exclusion criteria. Not only demographic characteristics such as age, sex, and handedness will be listed in the including criteria, but also factors which possibly influencing cerebral activities, including the drinking coffee or abusing alcohol and drugs, will be taken into consideration. Furthermore, in order to reduce the influence of emotional state on brain activities of patients with KOA, the SAS and SDS will be used to evaluate the patients’ psychological states. Second, at the stage of treatment, the placebo capsule is identical in appearance, odor, dosage, color, and texture to the celecoxib capsule. Third, at the stage of the fMRI scan, the patients will be asked to follow uniform instructions, wear earplugs, close their eyes, and remain relaxed and awake during the scan. The head of the participant will be placed in the head mask and a sponge will be inserted to strengthen the fixation of the head to eliminate possible head motion.

In summary, this fMRI trial is designed to investigate the central mechanism of celecoxib in the treatment of KOA by comparing the cerebral responses to celecoxib, placebo, and waiting list and by analyzing the correlation between the cerebral activity changes and clinical variables’ improvement. We expect that our findings can provide a reference for the clinical application of NSAID.

### Trial status

The trial is currently in the participant recruitment stage. The trial began recruitment on October 18, 2017 and is expected to be complete on December 31, 2019. This trial is reported in the light of the Standard Protocol Items: Recommendations for Intervention Trials (SPIRIT) guidelines (Additional file 1), [38].

## Additional file

Additional file 1: SPIRIT 2013 checklist. (DOC 136 kb)

## Abbreviations

ACR: American College of Rheumatology
ATS: Attention Test Scale
CNS: Central nervous system
COX: Cyclooxygenase
FC: Functional connectivity
fMRI: Functional magnetic resonance imaging
KOA: Knee osteoarthritis
MRI: Magnetic resonance imaging
NSAID: Non-steroidal anti-inflammatory drug
PAS: Pain Assessment of Sphygmomanometer
PG: Prostaglandin
ReHo: Regional homogeneity
rs-fMRI: Resting-state functional magnetic resonance imaging
SAS: Self-rating Anxiety Scale
SDS: Self-rating Depression Scale
SF-12: 12-Item Short Form Health Survey
TCM: Traditional Chinese medicine
TNF-α: Tumor necrosis factor-alpha
VAS: Visual Analog Scale
WOMAC: Western Ontario and McMaster osteoarthritis index
